# Cell-free plasma hypermethylated *CASZ1*, *CDH13* and *ING2* are promising biomarkers of esophageal cancer

**DOI:** 10.7555/JBR.32.20170065

**Published:** 2018-08-02

**Authors:** Huan-qiang Wang, Cong-ying Yang, Si-yuan Wang, Tian Wang, Jing-ling Han, Kai Wei, Fu-cun Liu, Ji-da Xu, Xian-zhen Peng, Jian-ming Wang

**Affiliations:** 1 Department of Public Health and Preventive Medicine, Kangda College of Nanjing Medical University, Lianyungang, Jiangsu 222000, China; 2 Department of Clinical Medicine, Kangda College of Nanjing Medical University, Lianyungang, Jiangsu 222000, China; 3 Department of Epidemiology, School of Public Health, Nanjing Medical University, Nanjing, Jiangsu 211166, China; 4 Department of Social Medicine and Health Education, School of Public Health, Nanjing Medical University, Nanjing, Jiangsu 211166, China; 5 The Innovation Center for Social Risk Governance in Health, School of Public Health, Nanjing Medical University, Nanjing, Jiangsu 211166, China; 6 Department of Pathology, The First People's Hospital of Lianyungang, Lianyungang, Jiangsu 222000, China

**Keywords:** esophageal neoplasms, DNA methylation, epigenesist, biological markers, tumor suppressor

## Abstract

Identifying sensitive and specific biomarkers for early detection of cancer is immensely imperative for early diagnosis and treatment and better clinical outcome of cancer patients. This study aimed to construct a specific DNA methylation pattern of cancer suppressor genes and explore the feasibility of applying cell-free DNA based methylation as a biomarker for early diagnosis of esophageal squamous cell carcinoma (ESCC). We recruited early stage ESCC patients from Yangzhong County, China. The Illumina Infinium 450K Methylation BeadChip was used to construct a genome-wide DNA methylation profile. Then, differentiated genes were selected for the validation study using the Sequenom MassARRAY platform. The frequency of methylation was compared between cancer tissues, matched cell-free DNAs and normal controls. The specific methylation profiles were constructed, and the sensitivity and specificity were calculated. Seven CG sites in three genes* CASZ1*, *CDH13* and *ING2* were significantly hypermethylated in ESCC as compared with normal controls. A significant correlation was found between the methylation of DNA extracted from cancer tissues and matched plasma cell-free DNA, either for individual CG site or for cumulative methylation analysis. The sensitivity and specificity reached 100% at an appropriate cut-point using these specific methylation biomarkers. This study revealed that aberrant DNA methylation is a promising biomarker for molecular diagnosis of esophageal cancer. Hypermethylation of *CASZ1*,* CDH13* and* ING2* detected in plasma cell-free DNA can be applied as a potential noninvasive biomarker for diagnosis of esophageal cancer.

## Introduction

Esophageal cancer is the eighth most common cancer in terms of incidence and ranks sixth in cancer-related deaths worldwide^[1^–^2]^. It usually occurs in the middle or upper one-third of the esophagus as the squamous cell carcinoma, or in the lower one-third or junction of the esophagus and stomach as the adenocarcinoma. While esophageal adenocarcinoma has markedly increased in developed countries during last few decades, esophageal squamous cell carcinoma (ESCC) still accounts for a large part, especially in Asian countries^[3]^. In spite of the advances in diagnostic methods and the improvements in surgical techniques and adjuvant chemo-radiation therapy, the majority of ESCC are diagnosed at advanced stages with the overall 5-year survival rates of 15%–50%^[4^–^5]^. If patients can be diagnosed and treated at an early stage, the 5-year survival rate would reach to 100% after the endoscopic mucosectomy^[6]^. Therefore, to identify sensitive and specific biomarkers for the early detection of this disease is immensely imperative for the patient’s prognosis.

Carcinogenesis of esophageal cancer is a multifactorial process by the accumulation of heritable changes in both oncogenes and tumor suppressor genes^[7]^. Cancer-related genes may be changed by several genetic mechanisms, which potentially alter the protein encoding nucleotide template, or change the copy number of genes. Epigenetic alteration, comprises heritable changes in gene expression that are not caused by alterations of DNA sequence^[8]^. These epigenetic alterations involve covalent modifications of histones and methylation of cytosine base (C) in the context of CpG dinucleotide within the DNA itself. Epigenetic changes usually occurs early preceding the malignancy^[9]^. A large-scale analysis of DNA methylation profiles could provide a better understanding of the molecular pathways involved in the esophageal carcinogenesis^[10^–^12]^. Several tumor suppressor genes, including* CDKN2A*^[13^–^15]^, *MGMT*^[13^,^16]^, *APC*^[17]^, *CD-H1*^[13]^,* DAPK*^[13]^, *FHIT*^[18]^, *RASSF1A*^[19]^, *CDH13*^[20]^, *BRCA1*^[13]^, *CHFR*^[21]^ and *SST*^[22]^ have been reported as hypermethylated in ESCC. As the pattern of DNA methylation has tumor specificity, we assume that discovering aberrant methylated genes and constructing specific profiles can identify potential biomarkers for the early diagnosis of ESCC.

Traditional methods used for detecting DNA methylation require tissue samples, which have limited their clinical applications. Free circulating DNA, an extracellular DNA widely existing in the plasma or serum of cancer patients, has gained attention recently. Studies have provided concrete evidence that cell-free DNA is released by tumors into the circulation^[23]^. It has consistent genetic characteristics and epigenetic changes with tumor cells, making it be an alternative promising biomarker^[24]^. The biology and diagnostic applications of cell-free DNA has attracted more and more interests for its minimally invasive, convenient and easily accepted properties^[25]^. Aberrant methylation cell-free DNA detected in the blood circulation appears to be a better biomarker for human cancers^[26]^.

This study aimed to compare the consistency of DNA methylation between esophageal cancer tissues and cell-free DNA extracted from the plasma of ESCC patients, construct DNA methylation patterns of cancer suppressor genes and explore the feasibility of applying cell-free DNA based methylation profile as a biomarker for early diagnosis of esophageal cancer.

## Subjects and methods

### Subjects

**Fig.1:**
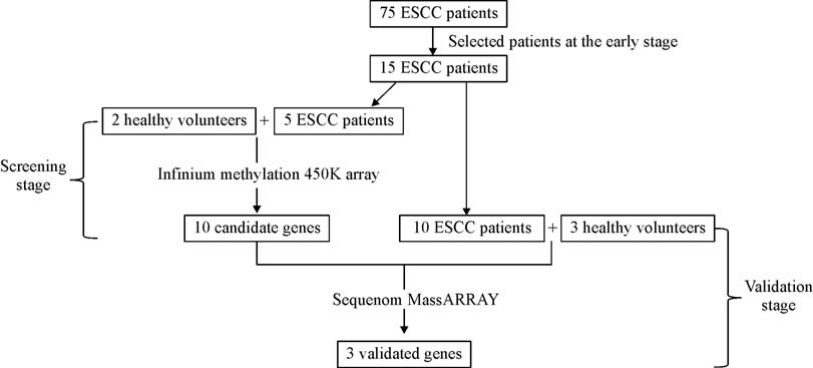
Flow chart of the study.

This study was performed in Yangzhong, an island county in the middle of Yangtze River in the southeast part of Jiangsu Province of China. It is well known for its high mortality and incidence of both stomach and esophageal cancers^[27]^. We recruited 75 esophageal cancer cases who underwent surgery at Yangzhong People’s Hospital between 2012 and 2013. ESCC was pathologically diagnosed in the patients. None of the enrolled patients had received preoperative chemotherapy or radiation therapy. The tumor stage was determined using the Classification of Malignant Tumours Staging System (TNM). Among them, we selected 15 patients during the early stage of ESCC as the study subjects (***Supplementary Table 1***, available online, and ***Table 1***). The flow chart of the study is in ***Fig. 1***. This study was approved by the ethics committee of Nanjing Medical University. The methods were carried out in accordance with the approved guidelines and a written informed consent was obtained from all participants.

**Tab.1:** Demographic and baseline information of esophageal squamous cell carcinoma patients in the current study

Patient No.	Age (years)	Gender	TNM	G (histologic grade)	Tumor location	Stage*
1	69	Male	T2N0M0	G1	Middle	Ⅰ
2	47	Male	T1N1M0	G2	Lower	Ⅱ
3	69	Male	T1N0M0	G2	Lower	Ⅰ
4	48	Male	T3N0M0	G2	Middle	Ⅱ
5	63	Male	T1N0M0	G3	Middle	Ⅰ
6	72	Female	T2N0M0	G1	Middle	Ⅰ
7	69	Male	T1N0M0	G3	Lower	Ⅰ
8	63	Female	T1N0M0	G2	Middle	Ⅰ
9	64	Male	T3N0M0	G2	Middle	Ⅱ
10	62	Male	T2N0M0	G1	Middle	Ⅰ

*Tumor stages were evaluated according to the TNM classification of the American Joint Committee on Cancer (AJCC).

### Data collection

Peripheral blood samples were acquired with vacuum blood tubes prior to the treatment. The plasma samples were immediately isolated by centrifugation at 4,000 r/minute for 10 minutes and stored at –70 °C until the DNA was extracted. For patients having undergone surgery, tissues in the center of the cancer lesion and remote normal appearing esophagus were excised and stored in –70 °C refrigerator immediately. Normal esophageal tissues together with blood samples were collected from healthy individuals who took part in the local health examination program and proved to have no cancer history and severe esophageal lesions by endoscopy. All healthy subjects provided informed consent to participate in the study.

### DNA extraction

Genomic DNA was extracted from frozen fresh tissues using the QIAmp DNA Mini Kit (Qiagen, Hilden, Germany) according to the manufacturer’s instructions. Cell-free DNA was isolated from the 200 μL plasma samples using the QIAmp DNA Blood Mini Kit (Qiagen, Hilden, Germany). The quality and concentration of DNA samples were measured with a Thermo NanoDrop 2000-1 spectrophotometer (NanoDrop Technologies, Montchanin, DE, USA).

### DNA methylation assay

DNAs were subjected to sodium bisulphite modification using the EZ DNA Methylation kit (Zymo Research, Orange, CA, USA). We measured the aberrant methylation status of DNA samples from ESCC patients based on a two-stage approach. Firstly, we used the Illumina Infinium 450K Methylation BeadChip (Illumina, San Diego, CA, USA) to construct a genome-wide DNA methylation profile. Secondly, differentiated CpG sites were screened by comparing the methylation of cancer and normal tissues. Then, we used the Sequenom MassARRAY platform to perform a quantitative analysis of DNA methylation for specific genes identified from the first stage analysis. This system utilized the base-specific cleavage and matrix-assisted laser desorption/ionization time-of-flight mass spectrometry (MALDI-TOF MS). In brief, after sodium bisulphite modification of DNA samples, the target gene regions were amplified by the Polymerase Chain Reaction (PCR). Primers were derived from Sequenom EpiDesigner platform (www.epidesigner.com) (***Table 2***). Considering the fragmentation of cell-free DNA, we designed multiple primers to detect methylation of specific sites for plasma samples (***Table 3***). Subsequently, unincorporated dNTPs left over were neutralized by dephosphorylation using the shrimp alkaline phosphatase. By tagging the reverse PCR primer with the T7 recognition sequence, a single-stranded RNA copy of the template was generated by *in vitro* transcription. After base specific (U-specific) cleavage by RNase A, the cleavage products were analyzed using MassARRAY. Signals with a 16 Da shift were representative for methylation events, and signal intensity was correlated with the degree of DNA methylation^[28]^. The methylation status ranged from 0 (completely unmethylated) to 1 (completely methylated).

**Tab.2:** Primers designed for detecting DNA methylation in tissues in the current study

Genes	Forward (5'→3')	Reverse (5'→3')
*ADAMTS9*	aggaagagagTATTTTGGGAAGTTGTGGTAGGTAG	cagtaatacgactcactatagggagaaggctCTCAACAAAAAAATCACCTTCTAACA
*AIM2*	aggaagagagTGTAGGTTTAGGTTTTTAGAGGTGTG	cagtaatacgactcactatagggagaaggctAAAACTCCAATTATCACTCCTACCC
*CASZ1*	aggaagagagTAGGGTATGTGGTGATTTTGTTAGG	cagtaatacgactcactatagggagaaggctAAAACTCAAACCACTATACAAACCAA
*CDH13*	aggaagagagTTAGTTTTGTTTGGGTTATGGAAAA	cagtaatacgactcactatagggagaaggctCAAAAACAAACTACCTTTCAACTCC
*EBF3*	aggaagagagTTTTTAAAGGATATTTTGGGGTTTT	cagtaatacgactcactatagggagaaggctCCCCCTTAAAAAAAACTTATCAATC
*ING2*	aggaagagagTTTTGAATTTTTGATTTTAGGGGAT	cagtaatacgactcactatagggagaaggctACCTTCAAAAAAATATAAAAACCTACAA
*IQGAP2*	aggaagagagAGAATTGAAGTATGGGTATTGTTAAG	cagtaatacgactcactatagggagaaggctCCTCTACATTTCCTTTCAATAAAACA
*KLF6*	aggaagagagGGTTTGGAGTTTTTTTGTTTTTTTT	cagtaatacgactcactatagggagaaggctATTCATCACCCTAACTTCCTCCTAA
*TMEFF2*	aggaagagagGAAAATTTTGATTGTGGGTTGTTA	cagtaatacgactcactatagggagaaggctCTTAAAAAACTCTCAAAAAACCAAT
*TRIT1*	aggaagagagGTTGTTTAGGTTTGGAGTGTAATGG	cagtaatacgactcactatagggagaaggctAATACCTAATACCCAAAAAACACCC

**Tab.3:** Primers designed for detecting cell-free DNA methylation in plasma cell-free DNA in the current study

Genes	Fragments	Forward (5'→3')	Reverse (5'→3')
*ADAMTS9*	ADAMTS9-17R	aggaagagagTGGTTTTAGTGTAGGATATTTTGAGTTT	cagtaatacgactcactatagggagaaggctCACTCCAACCTAAATAAAAAAATAAACC
	ADAMTS9-3F	aggaagagagGTTTTAGTTATTTGGGAGTTTGAGGT	cagtaatacgactcactatagggagaaggctAAACAAAAACTTACTCTCTCACCCA
*AIM2*	AIM2-5F	aggaagagagTTTGTAGGTTTAGGTTTTTAGAGGTG	cagtaatacgactcactatagggagaaggctTTATTTAAACATACTCTCCTAAATCCTCT
*CASZ1*		aggaagagagTAGGGTATGTGGTGATTTTGTTAGG	cagtaatacgactcactatagggagaaggctAAAACTCAAACCACTATACAAACCAA
*CDH13*	CDH13-17F	aggaagagagTTGTTTGGGAAGTAGAGTTGTTTTT	cagtaatacgactcactatagggagaaggctACTACCTTTCAACTCCAAATTCCTAA
	CDH13-3F	aggaagagagTTGGGTTATGGAAAATTTTGTATTTAT	cagtaatacgactcactatagggagaaggctACCCAACATTACCAAACAATACCTA
*EBF3*	EBF3-11R	aggaagagagGTTGTTTTTGATTTGTTAGGGAAAT	cagtaatacgactcactatagggagaaggctTCATCTTTTTAAACTCTAAATCAAATCC
	EBF3-1F	aggaagagagGTGGTTTTTTTGATAGGTTAGAGGT	cagtaatacgactcactatagggagaaggctTTAAAAACAAATTCCCAATATATACTCT
*ING2*		aggaagagagTTTTGAATTTTTGATTTTAGGGGAT	cagtaatacgactcactatagggagaaggctACCTTCAAAAAAATATAAAAACCTACAA
*IQGAP2*		aggaagagagAGAATTGAAGTATGGGTATTGTTAAG	cagtaatacgactcactatagggagaaggctCCTCTACATTTCCTTTCAATAAAACA
*KLF6*	KLF6-2F	aggaagagagGTTGGGGAAGGTAGGTTTAGTTTTA	cagtaatacgactcactatagggagaaggctAACATTACCCTCCCTAAATACATCA
*TMEFF2*	TMEFF2-9F	aggaagagagTTTTAATATTTTTTGGTTTGGGGTT	cagtaatacgactcactatagggagaaggctCTTAAAAAACTCTCAAAAAACCAATTC
	TMEFF2-9F	aggaagagagAAAATTTTGATTGTGGGTTGTTAAG	cagtaatacgactcactatagggagaaggctACACTAAAAAATTTTACTACCTCCCC
*TRIT1*	TRIT1-1	aggaagagagTTTGTTGTTTAGGTTTGGAGTGTAA	cagtaatacgactcactatagggagaaggctATCCCAACATATTAAAAAACCAAAA
	TRIT1-3F	aggaagagagGTTTTGGTTTTTTAATATGTTGGGA	cagtaatacgactcactatagggagaaggctAATACCCAAAAAACACCCAATACTT

### Statistical analysis

Data generated from the 450K DNA Methylation BeadChip were visualized and analyzed using the GenomeStudio Software version 2011.1 (Illumina). Each CpG site on the BeadChip was represented by two bead types representing the methylated (M) and unmethylated (U) state at that site. The methylation value for each CpG locus was expressed as a β value^[29]^. We calculated the diffScore by taking into account the background noise and sample variability. Individual CpG site was determined statistically significant based on two criteria: (1) absolute diffScore measured using the Illumina custom model and adjusted for the multiple comparisons was greater than 15; and (2) degree of difference measured by the changes in β value (Delta β) was greater than 0.31 (corresponding to an average of 31% or greater methylation difference). Delta β= (β_tumor_ -β_normal_). DiffScore=10 Sigh (β_tumor_ -β_normal_) log_10_P. Hierarchical unsupervised clustering analysis was used to draw heat maps of specific sites by comparing normal and tumor tissues. The pathway and function analysis were performed using KEGG and Gene Ontology (GO). Data generated from Sequenom MassARRAY platform were analyzed using IBM SPSS Statistics 18.0 (IBM Corp., NY, USA). We not only considered the methylation status of individual CpG site, but also calculated the cumulative methylation of multiple CpG sites in specific genes using additive models. The correlation of methylation between tumor tissues and matched plasma samples was analyzed using correlation analysis.

## Results

### Identification of differentially methylated CpG sites

Using the Infinium Methylation 450K array, we evaluated the methylation status of five paired tumor samples and corresponding adjacent normal-appearing tissues from ESCC patients, along with two normal esophageal tissue samples from the healthy population. A total of 173 CpG sites showed statistical significance with the absolute diffScore≥15 and Delta β≥0.31 between ESCC and normal esophageal tissue samples. Then we sorted CpG sites by the diffScore and defined another 200 CpGs. After removing duplications, a total of 354 CpG sites remained for analysis. We drafted a heat map illustrating the cluster of these 354 CpG sites in our previous study^[30]^. The heat map compared the methylation between tumor and normal tissues and showed a significantly differentiated pattern. However, in our previous study, we only focused on the methylation of promoter regions. In combination with the KEGG pathway and GO analysis, we identified ten genes in the other regions for the next-step validation study (***Table 4***). The KEGG pathway analysis showed that IQGAP2 and TRIT1 were involved in the regulation of actin cytoskeleton and metabolic pathways, respectively. The functions of these ten genes based on GO analyses were listed in ***Supplementary Table 2*** (available online).

**Tab.4:** The information of selected genes

Genes	Chromosome location	Start	Stop	Length (kb)	Position
*ADAMTS9*	3p14.3-p14.2	64598534	64770557	172024	3’UTR
*AIM2*	1q22	159032275	159046647	14373	5’UTR
*CASZ1*	1p36.22	10696661	10856707	160047	3’UTR
*CDH13*	16q24.2-q24.3	82660578	83830201	1169624	INTRON
*EBF3*	10q26.3	131633547	131762091	128545	INTRON
*ING2*	4q35.1	184426220	184432249	6030	INTRON
*IQGAP2*	5q13.3	75699149	76003957	304809	INTRON
*KLF6*	10p15	3821234	3827455	6222	3’UTR
*TMEFF2*	2q32.3	192814743	193059644	244902	INTRON
*TRIT1*	1p35.3-p34.1	40306703	40349177	42475	TSS1500

### Validation of methylation levels of specific genes

We further validated the methylation levels of specific CpG sites of these ten genes by comparing 10 ESCC tissues, 10 matched peripheral plasma samples and 3 normal esophageal tissues from healthy individuals. Individual CpG site analysis showed that CASZ1_ CpG_2.3, CASZ1_CpG_4, CASZ1_CpG_5, CDH13_CpG_8, ING2_CpG_1.2.3.4, ING2_CpG_5 and ING2_CpG_6.7 were significantly hypermethylated in ESCC tissues as compared with controls (***Table 5***). The cumulative effect analyses by considering multiple CpG sites in each gene also demonstrated an obvious difference of methylation levels between normal and cancer samples (CASZ1: *P* 0.001, CDH13: *P* 0.001, ING2: *P* 0.001) (***Table 6***). Interestingly, the similar difference was also observed in ESCC plasma as compared with controls (***Table 5*** and ***Table 6***). Moreover, the frequency of methylation in individual CpG site or the cumulative methylation in each gene was higher in plasma samples than that detected in cancer tissue samples.

**Tab.5:** Methylation of individual CG sites in specific genes in esophageal cancer patients and normal controls

CG sites	Methylation [%, mean(95%CI)]			*P* value (plasma *vs.* control)	*P* value (tissue *vs.* control)
	Plasma (*n*=10)	Tissue (*n*=10)	Control (*n*=3)		
***CASZ1***					
*CASZ1_CpG_2.3*	95.00(93.88–96.12)	88.20(84.95–91.45)	73.33(55.71–90.96)	0.001	0.001
*CASZ1_CpG_4*	83.40(77.21–89.59)	76.60(69.26–83.94)	48.33(37.13–59.53)	0.001	0.001
*CASZ1_CpG_5*	78.60(72.36–84.84)	71.10(65.63–76.57)	50.33(33.79–66.87)	0.001	0.001
***CDH13***					
*CDH13_CpG_8*	98.00(96.35–99.65)	77.80(66.50–89.10)	37.00(19.61–54.39)	0.001	0.001
***ING2***					
*ING2_CpG_1.2.3.4*	93.80(89.65–97.95)	52.60(36.36–68.84)	21.00(1.28–40.72)	0.011	0.001
*ING2_CpG_5*	83.80(69.86–97.74)	55.30(39.64–70.96)	24.67(8.50–40.83)	0.046	0.001
*ING2_CpG_6.7*	95.50(92.88–98.12)	60.80(45.21–76.39)	28.00(6.34–49.66)	0.006	0.001

**Tab.6:** Cumulative methylation of specific genes in esophageal cancer patients and control subjects

Genes	Cumulative methylation [mean(95%CI)]			*P* value (plasma *vs.* control)	*P *value (tissue *vs.* controls)
	Plasma (*n*=10)	Tissue (*n*=10)	Control (*n*=10)		
*CASZ1*	4.21(4.02–4.40)	3.88(3.56–4.20)	2.97(2.38–3.55)	0.001	0.001
*CDH13*	3.20(2.82–3.58)	2.32(1.82–2.82)	1.27(0.62–1.93)	0.024	0.001
*ING2*	2.73(2.60–2.86)	1.69(1.21–2.16)	0.74(0.17–1.31)	0.009	0.001

* Cumulative methylation was calculated by summing up the frequency of individual CpG sites in each gene.

### Correlation of methylation between tumor tissue and plasma sample

**Fig.2:**
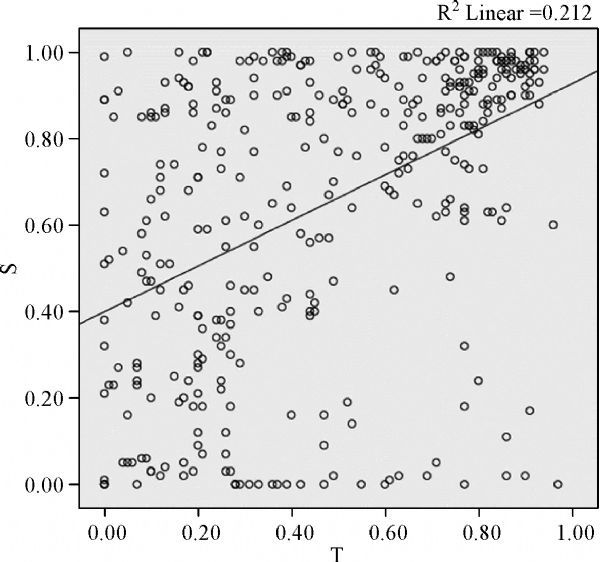
Scatter plot of individual CG site methylation in esophageal cancer tissue and plasma cell-free DNA.

**Fig.3:**
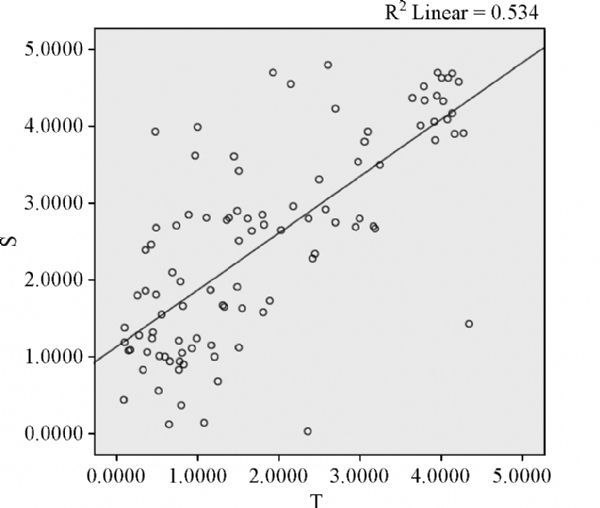
Scatter plot of cumulative methylation detected in esophageal cancer tissue and plasma cell-free DNA.

A significant correlation was found between the methylation of DNA extracted from esophageal cancer tissues and cell-free DNA extracted from the matched plasmas, either for individual CpG site analysis (*r*=0.460, *P* 0.001, ***Fig. 2***) or for cumulative effect analysis on multiple CpG sites in each specific gene (*r*=0.730, *P* 0.001, ***Fig. 3***).

### Evaluation of circulating cell-free DNA methylation for diagnosing ESCC

To assess the clinical usefulness of plasma cell-free DNA methylation as diagnostic biomarkers for ESCC, we calculated the sensitivity and specificity together with the receiver operating characteristic (ROC) curve. If we adopted the above-mentioned seven CpG sites in three cancer specific genes, the sensitivity and specificity were all reached to100% at an appropriate cut-point (***Table 7*** and ***Supplementary Table 3***, available online). The area under the curve (AUC) of ROC was 1 either using the individual CpG site or cumulative methylation of multiple CpG sites in specific genes.

**Tab.7:** Diagnostic information of individual CG site methylation in cell-free plasma DNA

CG site	Cut-point	Sensitivity (%)	Specificity (%)	Correctly classified (%)	CG site	Cut-point	Sensitivity (%)	Specificity (%)	Correctly classified (%)
*CASZ1_CPG_23*	0.67	100.00	0.00	76.92	*ING2_CPG_1234*	0.12	100.00	0.00	76.92
	0.72	100.00	33.33	84.62		0.24	100.00	33.33	84.62
	0.81	100.00	66.67	92.31		0.27	100.00	66.67	92.31
	0.92	100.00	100.00	100.00		0.86	100.00	100.00	100.00
	0.93	80.00	100.00	84.62		0.89	60.00	100.00	69.23
	0.94	70.00	100.00	76.92		0.94	50.00	100.00	61.54
	0.95	50.00	100.00	61.54		0.96	40.00	100.00	53.85
	0.96	10.00	100.00	30.77		0.99	20.00	100.00	38.46
	0.97	0.00	100.00	23.08		1.00	0.00	100.00	23.08
*CASZ1_CPG_4*	0.44	100.00	0.00	76.92	*ING2_CPG_5*	0.18	100.00	0.00	76.92
	0.48	100.00	33.33	84.62		0.25	100.00	33.33	84.62
	0.53	100.00	66.67	92.31		0.31	100.00	66.67	92.31
	0.63	100.00	100.00	100.00		0.32	100.00	100.00	100.00
	0.73	80.00	100.00	84.62		0.73	80.00	100.00	84.62
	0.83	70.00	100.00	76.92		0.87	70.00	100.00	76.92
	0.86	60.00	100.00	69.23		0.88	60.00	100.00	69.23
	0.87	40.00	100.00	53.85		0.89	50.00	100.00	61.54
	0.88	20.00	100.00	38.46		0.90	30.00	100.00	46.15
	0.89	0.00	100.00	23.08		0.93	0.00	100.00	23.08
*CASZ1_CPG_5*	0.46	100.00	0.00	76.92	*ING2_CPG_67*	0.18	100.00	0.00	76.92
	0.47	100.00	33.33	84.62		0.32	100.00	33.33	84.62
	0.58	100.00	66.67	92.31		0.34	100.00	66.67	92.31
	0.63	100.00	100.00	100.00		0.86	100.00	100.00	100.00
	0.65	80.00	100.00	84.62		0.93	80.00	100.00	84.62
	0.75	70.00	100.00	76.92		0.96	50.00	100.00	61.54
	0.76	60.00	100.00	69.23		0.97	30.00	100.00	46.15
	0.83	20.00	100.00	38.46		0.98	0.00	100.00	23.08
	0.87	10.00	100.00	30.77					
	0.88	0.00	100.00	23.08					
*CDH13_CPG_8*	0.29	100.00	0.00	76.92					
	0.40	100.00	33.33	84.62					
	0.42	100.00	66.67	92.31					
	0.92	100.00	100.00	100.00					
	0.98	90.00	100.00	92.31					
	1.00	30.00	100.00	46.15					

## Discussion

Along with the genetic variants, aberrant methylation is another predominant mechanism inactivating tumor suppressor genes during the carcinogenesis. In this study, we demonstrate a specific DNA methylation pattern of tumor-related genes for ESCC. The consistency of methylation between cancer tissues and plasma indicates that cell-free DNA methylation profile can be a promising biomarker for the diagnosis of esophageal cancer.

We used the Infinium Human Methylation 450K BeadChip to screen for differentiated CpG sites between esophageal cancer and normal control tissues. This BeadChip allows researchers to interrogate more than 485,000 methylation sites per sample at single-nucleotide resolution. It has emerged as one of the preferred methodologies to study genome-wide DNA methylation because of its optimal combination of genome-wide coverage (96% of CpG islands and 99% of RefSeq genes), comprehensive representation of functional gene sub-regions, and good reproducibility across other platforms, and relative affordability^[31]^.

Based on the genome-wide DNA methylation data, 10 specific tumor suppressor genes were selected for the validation study. Among them, seven CpG sites from three genes (*CASZ1*, *CDH13* and *ING2*) were significantly hypermethylated in ESCC tissues. *CASZ1* is a zinc finger transcription factor which is critical for controlling the normal differentiation of subtypes of neural and cardiac muscle cells^[32]^. It inhibits cell cycle progression in neuroblastoma by restoring pRb activity^[33]^. Loss of *CASZ1* function is associated with a poor prognosis of the disease^[32]^. *CDH13*, a member of the cadherin gene superfamily has been mapped to 16q24, a locus that frequently undergoes deletion in human cancers^[20]^. As a candidate tumor suppressive gene, ING2 is frequently decreased expression in human tumors^[34]^. Nonphysiological overexpression of *ING2* induces apoptosis and cell cycle arrest *via* p53 modification^[35]^. Aberrant methylation of these genes can alter their normal function, resulting in the carcinogenesis of ESCC^[36]^.

DNA methylation has been regarded as the most robust epigenetic mark. Almost any biological tissue sample or body fluid can be used for analyzing DNA methylation^[37]^. Tumor biopsies can be sampled in most cancers but are often difficult to obtain. For the early detection of cancers, only peripheral, easy-to-access tissues or bodily fluids can be collected. These samples include peripheral blood, saliva, urine, stools or bronchial aspirates, etc^[37^–^38]^.

Cell-free circulating tumor DNA is a potential surrogate for the entire tumor genome^[39]^. It has been estimated that more than 90% of the total circulating cell-free DNA is derived from the tumor tissue^[40]^. The release of nucleic acids into the blood is thought to be related to the apoptosis and necrosis of cancer cells in the tumor microenvironment^[26]^. Changes in the levels of circulating nucleic acids are associated with tumor burden and malignant progression. The presence of methylated DNA in the serum or plasma have been found in various types of malignancy, including bladder cancer, breast cancer, cervical cancer, colorectal cancer, lung cancer, and prostate cancer^[41]^.

Interestingly, cell-free circulating DNA methylation profiles are in high concordance with patterns observed in corresponding primary tumor tissues^[29^,^42]^. It has been linked to different types of cancer^[17^,^29^,^43]^. In our study, we confirmed a significant correlation of methylation patterns of tumor suppressor genes between plasma samples and matched tumor tissues. We could find that the r for cumulative effect analysis on multiple CpG sites in each specific gene was high for the individual CpG site analysis. And the fact that sites separated by less than 1 kb show highly correlated methylation^[44^–^46]^, suggesting that our analysis of cumulative effect on multiple CpG sites in each specific gene may capture more accurate methylation information near transcription start sites. Moreover, we observed that the methylation level of specific genes detected in plasma samples was higher than that in tumor tissues. This may be attributed to the heterogeneous mix of different cell types in the tumor tissues^[47]^. An accurate correlation of molecular and morphologic pathologies requires the ability to procure pure populations of morphologically similar cells for molecular analysis. Several approaches have been developed to address this problem, with laser capture microdissection (LCM) emerging as one of the methods of choice for isolating highly pure cells from a heterogeneous tissue section^[48]^. However, the invasive feature of tissue biopsy has restricted its clinical use. In this case, the blood-based assay has the advantage of using specimens which have been collected with a far less invasive procedure.

Despite the possible use as a marker for cancer diagnosis or progression, the limited quantities of cell-free DNA in the plasma affected its wide application. An increasing tendency to harmonize appropriate methods for DNA methylation detection and reference standards will accelerate the development of DNA methylation as biomarkers for cancer detection. Though several techniques such as MethyLight qPCR, MethyLight digital PCR (dPCR), methylation-sensitive and-dependent restriction enzyme (MSRE/MDRE) digestion followed by qPCR or dPCR, and bisulfite amplicon next generation sequencing (NGS) have been developed, there is currently a lack of consensus regarding the optimal methodologies to quantify methylation status^[49]^. Considering the fragmentation of cell-free DNA and the degradation by sodium bisulphite modification, a more sensitive and specific method is essential for clinical use of cell-free DNA methylation detection in the future.

In conclusion, aberrant methylated DNAs can be promising and valuable biomarkers for molecular diagnosis of esophageal cancer. Hypermethylation of *CASZ1*, *CDH13* and *ING2 *genes detected using cell-free DNA in blood samples can be applied as a potential biomarker for the diagnosis of esophageal cancer.

## References

[1] Zhang Y. Epidemiology of esophageal cancer[J]. World J Gastroenterol, 2013, 19(34): 5598–5606 . 24039351 10.3748/wjg.v19.i34.5598 PMC3769895

[2] Pennathur A, Gibson MK, Jobe BA, Oesophageal carcinoma[J]. Lancet, 2013, 381(9864): 400–412 . 23374478 10.1016/S0140-6736(12)60643-6

[3] Vizcaino AP, Moreno V, Lambert R, Time trends incidence of both major histologic types of esophageal carcinomas in selected countries, 1973–1995[J]. Int J Cancer, 2002, 99(6): 860–868 . 12115489 10.1002/ijc.10427

[4] Rutegård M, Charonis K, Lu Y, Population-based esophageal cancer survival after resection without neoadjuvant therapy: an update[J]. Surgery, 2012, 152(5): 903–910 . 22657730 10.1016/j.surg.2012.03.025

[5] van Hagen P, Hulshof MC, van Lanschot JJ, Preoperative chemoradiotherapy for esophageal or junctional cancer[J]. N Engl J Med, 2012, 366(22): 2074–2084 . 22646630 10.1056/NEJMoa1112088

[6] Wang GQ. 30-year experiences on early detection and treatment of esophageal cancer in high risk areas[J]. Zhongguo Yi Xue Ke Xue Yuan Xue Bao, 2001, 23(1): 69–72 . 12905824

[7] Sandoval J, Esteller M. Cancer epigenomics: beyond genomics[J]. Curr Opin Genet Dev, 2012, 22(1): 50–55 . 22402447 10.1016/j.gde.2012.02.008

[8] GrØnbaek K, Hother C, Jones PA. Epigenetic changes in cancer[J]. APMIS, 2007, 115(10): 1039–1059 . 18042143 10.1111/j.1600-0463.2007.apm_636.xml.x

[9] Dejeux E, Audard V, Cavard C, Rapid identification of promoter hypermethylation in hepatocellular carcinoma by pyrosequencing of etiologically homogeneous sample pools[J]. J Mol Diagn, 2007, 9(4): 510–520 . 17690210 10.2353/jmoldx.2007.060209 PMC1975099

[10] Baba Y, Watanabe M, Baba H. Review of the alterations in DNA methylation in esophageal squamous cell carcinoma[J]. Surg Today, 2013, 43(12): 1355–1364 . 23291904 10.1007/s00595-012-0451-y

[11] Li JS, Ying JM, Wang XW, Promoter methylation of tumor suppressor genes in esophageal squamous cell carcinoma[J]. Chin J Cancer, 2013, 32(1): 3–11 . 22572016 10.5732/cjc.011.10381 PMC3845589

[12] Kaz AM, Grady WM. Epigenetic biomarkers in esophageal cancer[J]. Cancer Lett, 2014, 342(2): 193–199 . 22406828 10.1016/j.canlet.2012.02.036 PMC3395756

[13] Guo M, Ren J, House MG, Accumulation of promoter methylation suggests epigenetic progression in squamous cell carcinoma of the esophagus[J]. Clin Cancer Res, 2006, 12(15): 4515–4522 . 16899597 10.1158/1078-0432.CCR-05-2858

[14] Taghavi N, Biramijamal F, Sotoudeh M, p16INK4a hypermethylation and p53, p16 and MDM2 protein expression in esophageal squamous cell carcinoma[J]. BMC Cancer, 2010, 10: 138 . 20388212 10.1186/1471-2407-10-138 PMC2868052

[15] Hibi K, Taguchi M, Nakayama H, Molecular detection of p16 promoter methylation in the serum of patients with esophageal squamous cell carcinoma[J]. Clin Cancer Res, 2001, 7(10): 3135–3138 . 11595706

[16] Zhang L, Lu W, Miao X, Inactivation of DNA repair gene O6-methylguanine-DNA methyltransferase by promoter hypermethylation and its relation to p53 mutations in esophageal squamous cell carcinoma[J]. Carcinogenesis, 2003, 24(6): 1039–1044 . 12807758 10.1093/carcin/bgg062

[17] Kawakami K, Brabender J, Lord RV, Hypermethylated APC DNA in plasma and prognosis of patients with esophageal adenocarcinoma[J]. J Natl Cancer Inst, 2000, 92(22): 1805–1811 . 11078757 10.1093/jnci/92.22.1805

[18] Noguchi T, Takeno S, Kimura Y, FHIT expression and hypermethylation in esophageal squamous cell carcinoma[J]. Int J Mol Med, 2003, 11(4): 441–447 . 12632095

[19] Kuroki T, Trapasso F, Yendamuri S, Allele loss and promoter hypermethylation of VHL, RAR-beta, RASSF1A, and FHIT tumor suppressor genes on chromosome 3p in esophageal squamous cell carcinoma[J]. Cancer Res, 2003, 63(13): 3724–3728 . 12839965

[20] Jin Z, Cheng Y, Olaru A, Promoter hypermethylation of CDH13 is a common, early event in human esophageal adenocarcinogenesis and correlates with clinical risk factors[J]. Int J Cancer, 2008, 123(10): 2331–2336 . 18729198 10.1002/ijc.23804

[21] Shibata Y, Haruki N, Kuwabara Y, Chfr expression is downregulated by CpG island hypermethylation in esophageal cancer[J]. Carcinogenesis, 2002, 23(10): 1695–1699 . 12376479 10.1093/carcin/23.10.1695

[22] Jin Z, Mori Y, Hamilton JP, Hypermethylation of the somatostatin promoter is a common, early event in human esophageal carcinogenesis[J]. Cancer, 2008, 112(1): 43–49 . 17999418 10.1002/cncr.23135

[23] Chan KC, Jiang P, Chan CW, Noninvasive detection of cancer-associated genome-wide hypomethylation and copy number aberrations by plasma DNA bisulfite sequencing[J]. Proc Natl Acad Sci U S A, 2013, 110(47): 18761–18768 . 24191000 10.1073/pnas.1313995110 PMC3839703

[24] Hattori N, Ushijima T. Compendium of aberrant DNA methylation and histone modifications in cancer[J]. Biochem Biophys Res Commun, 2014, 455(1–2): 3–9 . 25194808 10.1016/j.bbrc.2014.08.140

[25] Nie K, Jia Y, Zhang X. Cell-free circulating tumor DNA in plasma/serum of non-small cell lung cancer[J]. Tumour Biol, 2015, 36(1): 7–9 . 25352029 10.1007/s13277-014-2758-3

[26] Li X, Zhou F, Jiang C, Identification of a DNA methylome profile of esophageal squamous cell carcinoma and potential plasma epigenetic biomarkers for early diagnosis[J]. PLoS One, 2014, 9(7): e103162 . 25050929 10.1371/journal.pone.0103162 PMC4106874

[27] Wang JM, Xu B, Hsieh CC, Longitudinal trends of stomach cancer and esophageal cancer in Yangzhong County: a high-incidence rural area of China[J]. Eur J Gastroenterol Hepatol, 2005, 17(12): 1339–1344 . 16292087 10.1097/00042737-200512000-00012

[28] Gaedcke J, Leha A, Claus R, Identification of a DNA methylation signature to predict disease-free survival in locally advanced rectal cancer[J]. Oncotarget, 2014, 5(18): 8123–8135 . 25261372 10.18632/oncotarget.2347 PMC4226671

[29] Zhai R, Zhao Y, Su L, Genome-wide DNA methylation profiling of cell-free serum DNA in esophageal adenocarcinoma and Barrett esophagus[J]. Neoplasia, 2012, 14(1): 29–33 . 22355271 10.1593/neo.111626 PMC3281939

[30] Peng X, Xue H, LÜ L, Accumulated promoter methylation as a potential biomarker for esophageal cancer[J]. Oncotarget, 2017, 8(1): 679–691 . 27893424 10.18632/oncotarget.13510 PMC5352188

[31] Yousefi P, Huen K, Aguilar Schall R, Considerations for normalization of DNA methylation data by Illumina 450K BeadChip assay in population studies[J]. Epigenetics, 2013, 8(11): 1141–1152 . 23959097 10.4161/epi.26037 PMC6242262

[32] Virden RA, Thiele CJ, Liu Z. Characterization of critical domains within the tumor suppressor CASZ1 required for transcriptional regulation and growth suppression[J]. Mol Cell Biol, 2012, 32(8): 1518–1528 . 22331471 10.1128/MCB.06039-11 PMC3318574

[33] Liu Z, Rader J, He S, CASZ1 inhibits cell cycle progression in neuroblastoma by restoring pRb activity[J]. Cell Cycle, 2013, 12(14): 2210–2218 . 23892435 10.4161/cc.25265 PMC3755071

[34] Larrieu D, Ythier D, Brambilla C, ING2 controls the G1 to S-phase transition by regulating p21 expression[J]. Cell Cycle, 2010, 9(19): 3984–3990 . 20890119 10.4161/cc.9.19.13208

[35] Li X, Kikuchi K, Takano Y. ING genes work as tumor suppressor genes in the carcinogenesis of head and neck squamous cell carcinoma[J]. J Oncol, 2011, 2011: 963614 . 21052543 10.1155/2011/963614 PMC2968421

[36] Adalsteinsson BT, Ferguson-Smith AC. Epigenetic control of the genome-lessons from genomic imprinting[J]. Genes (Basel), 2014, 5(3): 635–655 . 25257202 10.3390/genes5030635 PMC4198922

[37] Mikeska T, Craig JM. DNA methylation biomarkers: cancer and beyond[J]. Genes (Basel), 2014, 5(3): 821–864 . 25229548 10.3390/genes5030821 PMC4198933

[38] Belinsky SA, Palmisano WA, Gilliland FD, Aberrant promoter methylation in bronchial epithelium and sputum from current and former smokers[J]. Cancer Res, 2002, 62(8): 2370–2377 . 11956099

[39] Heitzer E, Ulz P, Geigl JB. Circulating tumor DNA as a liquid biopsy for cancer[J]. Clin Chem, 2015, 61(1): 112–123 . 25388429 10.1373/clinchem.2014.222679

[40] Jahr S, Hentze H, Englisch S, DNA fragments in the blood plasma of cancer patients: quantitations and evidence for their origin from apoptotic and necrotic cells[J]. Cancer Res, 2001, 61(4): 1659–1665 . 11245480

[41] Schwarzenbach H, Hoon DS, Pantel K. Cell-free nucleic acids as biomarkers in cancer patients[J]. Nat Rev Cancer, 2011, 11(6): 426–437 . 21562580 10.1038/nrc3066

[42] Radpour R, Barekati Z, Kohler C, Hypermethylation of tumor suppressor genes involved in critical regulatory pathways for developing a blood-based test in breast cancer[J]. PLoS One, 2011, 6(1): e16080 . 21283676 10.1371/journal.pone.0016080 PMC3025923

[43] Liggett TE, Melnikov AA, Marks JR, Methylation patterns in cell-free plasma DNA reflect removal of the primary tumor and drug treatment of breast cancer patients[J]. Int J Cancer, 2011, 128(2): 492–499 . 20473856 10.1002/ijc.25363 PMC2970661

[44] Fraser HB, Lam LL, Neumann SM, Population-specificity of human DNA methylation[J]. Genome Biol, 2012, 13(2): R8 . 22322129 10.1186/gb-2012-13-2-r8 PMC3334571

[45] Li Y, Zhu J, Tian G, The DNA methylome of human peripheral blood mononuclear cells[J]. PLoS Biol, 2010, 8(11): e1000533 . 21085693 10.1371/journal.pbio.1000533 PMC2976721

[46] Eckhardt F, Lewin J, Cortese R, DNA methylation profiling of human chromosomes 6, 20 and 22[J]. Nat Genet, 2006, 38(12): 1378–1385 . 17072317 10.1038/ng1909 PMC3082778

[47] Zanni KL, Chan GK. Laser capture microdissection: understanding the techniques and implications for molecular biology in nursing research through analysis of breast cancer tumor samples[J]. Biol Res Nurs, 2011, 13(3): 297–305 . 21444330 10.1177/1099800411402054

[48] Craven RA, Banks RE. Laser capture microdissection and proteomics: possibilities and limitation[J]. Proteomics, 2001, 1(10): 1200–1204 . 11721632 10.1002/1615-9861(200110)1:10<1200::AID-PROT1200>3.0.CO;2-Q

[49] Redshaw N, Huggett JF, Taylor MS, Quantification of epigenetic biomarkers: an evaluation of established and emerging methods for DNA methylation analysis[J]. BMC Genomics, 2014, 15(1): 1174. 25539843 10.1186/1471-2164-15-1174 PMC4523014

